# Characterization of Extracellular Vesicles from Human Saliva: Effects of Age and Isolation Techniques

**DOI:** 10.3390/cells13010095

**Published:** 2024-01-02

**Authors:** Lucia Reseco, Angela Molina-Crespo, Mercedes Atienza, Esperanza Gonzalez, Juan Manuel Falcon-Perez, Jose L. Cantero

**Affiliations:** 1 Laboratory of Functional Neuroscience, Pablo de Olavide University, 41013 Seville, Spain; lrescal@upo.es (L.R.); amolcre@upo.es (A.M.-C.); matirui@upo.es (M.A.); 2 CIBERNED, Centro de Investigación Biomédica en Red de Enfermedades Neurodegenerativas, Instituto de Salud Carlos III, 28029 Madrid, Spain; 3 Exosomes Laboratory, Center for Cooperative Research in Biosciences (CIC bioGUNE), Basque Research and Technology Alliance (BRTA), 48160 Derio, Spain; egonzalez@cicbiogune.es (E.G.); jfalcon@cicbiogune.es (J.M.F.-P.); 4 CIBEREHD, Centro de Investigación Biomédica en Red de Enfermedades Hepáticas y Digestivas, 28029 Madrid, Spain

**Keywords:** saliva, extracellular vesicles, exosomes, aging, TEM, magnetic bead immunocapture, tetraspanins

## Abstract

Salivary extracellular vesicles (EVs) represent an attractive source of biomarkers due to the accessibility of saliva and its non-invasive sampling methods. However, the lack of comparative studies assessing the efficacy of different EV isolation techniques hampers the use of salivary EVs in clinical settings. Moreover, the effects of age on salivary EVs are largely unknown, hindering the identification of salivary EV-associated biomarkers across the lifespan. To address these questions, we compared salivary EV concentration, size mode, protein concentration, and purity using eight EV isolation techniques before and after magnetic bead immunocapture with antibodies against CD9, CD63, and CD81. The effects of age on salivary EVs obtained with each isolation technique were further investigated. Results showed higher expression of CD63 on isolated salivary EVs compared to the expression of CD81 and flotillin-1. Overall, magnetic bead immunocapture was more efficient in recovering salivary EVs with Norgen’s Saliva Exosome Purification Kit and ExoQuick-TC ULTRA at the cost of EV yield. Regardless of age, Invitrogen Total Exosome Isolation Solution showed the highest level of protein concentration, whereas Izon qEVOriginal-70nm columns revealed the highest purity. This study provides the first comprehensive comparison of salivary EVs in younger and older adults using different EV isolation techniques, which represents a step forward for assessing salivary EVs as a source of potential biomarkers of tissue-specific diseases throughout the life cycle.

## 1. Introduction

Saliva is a remarkably complex fluid that fulfills a large number of functions such as the lubrication of the oral cavity, digestion, immunity, and overall maintenance of physiological homeostasis [1]. In recent years, saliva has gained considerable attention in the search of biomarkers due to its easy accessibility, non-invasive methods of collection, and fewer compliance problems. Moreover, the analysis of saliva represents a potentially cost-effective approach for the screening of large populations and performing follow-up studies [2]. Although saliva is about 99% water, it also contains electrolytes, peptides, proteins, glycoproteins, lipids, metabolites, messenger RNAs (mRNAs), microRNAs (miRNAs), and genomic DNA, which are actively secreted by salivary glands under direct autonomic innervation and release of major neurotransmitters [3,4]. Salivary glands are highly vascularized tissues, enabling the transport of blood molecules into saliva through different mechanisms, which will depend upon the physicochemical properties of molecules, size, and presence of transporters [5]. Consequently, a substantial portion of the salivary proteome is found in blood [6], opening up opportunities to use saliva as a preferred diagnostic fluid.

Extracellular vesicles (EVs) are a heterogeneous group of membrane-bound vesicles secreted by numerous cell types. The molecular content of EVs is heavily dependent on the tissue/cell type from which they are derived, mirroring the phenotypic and physiological state of donor cells [7]. EVs have been involved in intercellular communication through the exchange of proteins, nucleic acids, and lipids between cells [8], supporting their role in many physiological processes and pathological conditions [9]. Remarkably, EVs are present in nearly all types of biofluids, showing tremendous potential for liquid biopsy and becoming an unexpected source of biomarkers for a wide range of systemic diseases [10,11].

Accumulated evidence indicates that EVs are present in human saliva [12,13]; they are similar in morphology and size to EVs released in other body fluids [14] and are highly enriched in miRNAs [15,16,17], raising the possibility that they are suitable for non-invasive monitoring of pathological conditions. However, clinical research on salivary EVs is still in its infancy, partially due to the lack of systematic studies assessing the efficacy of different EV isolation methods in saliva. Up to now, only one study has compared the efficacy of two different methods to isolate salivary EVs in middle-aged adults [18]. Results revealed that ExoQuick-TC, a commonly used precipitation-based technique, isolated larger salivary exosomes and exhibited more biological impurities than differential ultracentrifugation (UC). However, this study neither assessed the performance of size exclusion chromatography (SEC) techniques nor evaluate the effects of magnetic bead recovery on EV concentration and size, which may modify the yield, purity, and morphology of salivary EVs. Moreover, the effects of age on salivary EVs remain poorly understood, hampering the identification and validation of salivary EV-based disease biomarkers across the lifespan.

In the present study, we compared EV concentration, size mode, protein concentration, and purity using eight techniques to isolate human salivary EVs before and after magnetic bead immunocapture with antibodies against CD9, CD63, and CD81. Our hypothesis was that EV isolation techniques based on SEC columns would show lower EV yield and lower protein concentration than those techniques based on precipitation reagents or differential UC. Evidence has shown that submandibular salivary glands, the major contributor to the unstimulated salivary flow rate [19], are more vulnerable to aging than parotid glands [20,21]. Therefore, we would expect the yield and size of salivary EVs to be reduced in older adults compared to their younger counterparts, likely suggesting age-related hypofunction of submandibular salivary glands, which may have practical implications for the use of salivary EVs as a source of potential biomarkers in aging-associated pathological conditions.

## 2. Methods

### 2.1. Subjects

Two experiments were conducted in the present study. The first experiment was aimed at comparing the effectiveness and purity of eight techniques for salivary EV isolation and further characterizing age-related changes in human salivary EVs. For this purpose, 6 collaborative volunteer donors, 3 younger (25, 28, 29 years, two women) and 3 older adults (58, 64, 66 years, two women), were recruited. We performed a second experiment to confirm age-related changes in salivary EVs at the group level by using the most effective EV isolation technique identified in the first experiment. The second experiment was conducted on 10 young (26 ± 1.9 years; age range: 24–29 years; 5 females) and 10 older adults (64.3 ± 3.2 years; age range: 60–70 years; 4 females).

Most of the younger participants were PhD students enrolled at Pablo de Olavide University. Older adults were recruited from senior citizen’s associations, health-screening programs, and hospital outpatient services. All participants underwent a medical history interview to discard chronic medical conditions such as heart disease, cancer, respiratory disease, gastrointestinal disorders, metabolic disease, stroke, genetic diseases, and/or other disorders affecting brain structure or function (e.g., cerebrovascular disease, epilepsy, head trauma, history of neurodevelopmental disease, alcohol abuse, hydrocephalus, and/or intracranial mass). None of them were taking medications that could affect cognition, sleep, renal, and/or hepatic function.

### 2.2. Saliva Collection

Fasting whole saliva samples were collected in the morning (9:00–10:00 AM). Participants were asked to refrain from any oral stimulation (e.g., food, drink, smoke, chew gum, oral hygiene) for about 8 h prior to saliva collection, and they were not allowed to wear lipstick or lip balm to avoid sample contamination. In the first experiment, younger adults provided 30, 32, and 31 mL of saliva, whereas older adults provided 21, 20, and 29 mL of saliva. For the second experiment, a minimum of 5 mL of saliva was obtained from each participant.

Saliva was collected using the spitting method. The choice of this technique was based on different criteria: (i) the simplicity of use in clinical and research contexts with people of all ages, as long as they are collaborative [22]; (ii) the best choice when the salivary flow rate is low, as in elderly participants, and when the evaporation of saliva has to be minimized due to long-time samplings [23]; and (iii) because it has shown the lowest individual variability when compared to other techniques employed to collect resting saliva [24].

Saliva samples were collected into 50 mL conical sterile polypropylene tubes previously treated with a 2% sodium azide solution, and the tubes were kept on ice during the collection process. The presence of blood contamination in saliva was excluded by visual inspection. After collection, samples were immediately placed on ice and cleared using differential centrifugation at 162× *g* (1000 rpm) for 10 min at 4 °C. The supernatant was then centrifuged at 2287× *g* (4500 rpm) for 15 min at 4 °C, aliquoted into 1.5 mL sterile polypropylene tubes with a protease inhibitor cocktail 1X (Complete Ultra Tablets Mini, Roche, Basel, Switzerland), and stored at −80 °C until EV isolation.

### 2.3. Salivary EV Isolation

To circumvent the issue of obtaining large volumes of saliva in each individual for the first experiment (i.e., 24 mL of saliva/participant = 500 µL of saliva × 8 EV isolation techniques × 6 replicates), saliva samples obtained from three participants per group of age were pooled in each of the six replicates conducted on different days. Each replicate (N = 6) resulted from mixing 1.5 mL of saliva from each participant until reaching 4 mL (500 µL of saliva × 8 EV isolation techniques = 4 mL).

**Figure 1:**
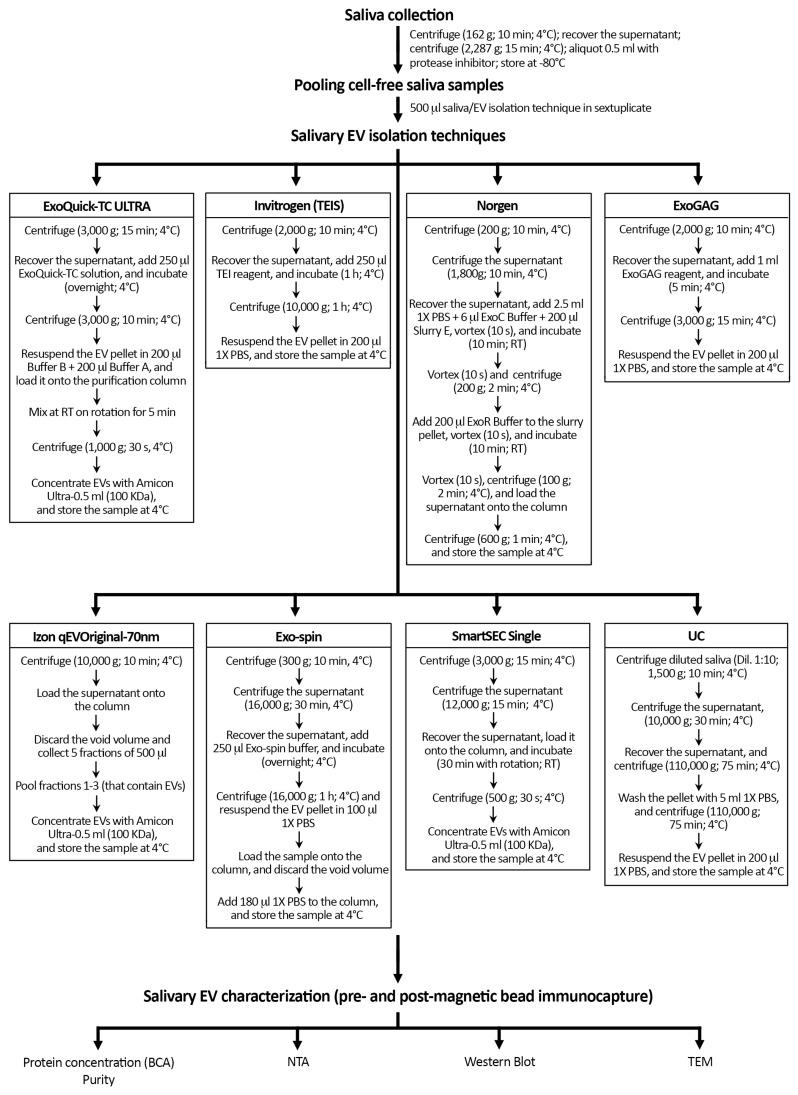
Schematic overview of the protocol followed with each technique for the isolation of salivary EVs. Salivary EV isolation was performed in sextuplicate with eight techniques in younger and older adults, respectively. Protein concentration and purity of salivary EV samples were only assessed before magnetic bead immunocapture with anti-CD9, -CD63, and -CD81 antibodies. TEIS: Total Exosome Isolation Solution; RT: room temperature; UC: differential ultracentrifugation; BCA: Bicinchoninic acid protein assay; NTA: nanoparticle tracking analysis; TEM: transmission electron microscopy.

Figure 1 shows a schematic overview of the protocol followed with each technique for the isolation of salivary EVs. Once thawed, we pooled 500 µL of cell-free saliva sample and mixed homogeneously by pipetting before EV isolation with each of the following 8 techniques: (i) ExoQuick-TC ULTRA (System Biosciences, Palo Alto, CA, USA), (ii) Invitrogen Total Exosome Isolation Solution (Thermo Fisher Scientific, Waltham, MA, USA), (iii) Norgen’s Saliva Exosome Purification Kit (NORGEN Biotek Corp., Thorold, ON, Canada), (iv) ExoGAG purification kit (Nasasbiotech, S.L., A Coruña, Spain), (v) Izon qEVOriginal-70nm (IZON Science, Oxford, UK), (vi) Exo-spin (Cell Guidance Systems, Cambridge, UK), (vii) SmartSEC Single (System Biosciences, Palo Alto, CA, USA), and (viii) differential UC.

Differential UC was performed in a Beckman Coulter Optima XE-100 system using a 70 Ti fixed-angle titanium rotor equipped with open-top tubes. To reduce the viscosity of the sample and increase the efficiency of EV isolation, 500 µL of saliva was topped up to 5 mL with 0.22 µm-filtered 1X PBS (Gibco, Grand Island, NY, USA). Diluted saliva samples were centrifuged at low speed (1500× *g* for 10 min at 4 °C) to remove dead cells and cell debris. The supernatant was first centrifuged (10,000× *g* for 30 min at 4 °C) to sediment microvesicles and apoptotic bodies and then ultracentrifuged at 110,000× *g* for 75 min at 4 °C. Finally, the resulting pellet was resuspended in 5 mL of 0.22 µm-filtered 1X PBS and centrifuged at 110,000× *g* for 75 min at 4 °C to reduce contamination by soluble proteins.

To concentrate EVs, one extra step was conducted with ExoQuick-TC ULTRA, Izon qEVOriginal-70nm, and SmartSEC Single techniques by using Amicon Ultra-0.5 mL 100 KDa centrifugal filters (Merck KGaA, Darmstadt, Germany). For further comparison, EVs obtained with each technique were diluted up to a final volume of 200 µL with 0.22 µm-filtered 1X PBS: 100 µL were used for characterization of salivary EVs and total protein quantification prior to magnetic bead immunocapture, and the remaining 100 µL were employed for exosome purification through magnetic bead immunocapture and further characterization. Samples were stored at 4 °C until EV characterization, which included nanoparticle tracking analysis (NTA), western blots (WB), and transmission electron microscopy (TEM).

### 2.4. Total Protein Quantification

Protein concentration was measured before magnetic bead immunocapture using the Pierce BCA Protein Assay Kit (Thermo Fisher Scientific, Waltham, MA, USA), according to the manufacturer’s instructions. The particle-to-protein ratio was further computed to estimate EV purity [25].

### 2.5. Magnetic Bead Immunocapture

Following EV isolation with each technique, salivary exosomes were captured on a DynaMag-2 separation rack (Thermo Fisher Scientific, Waltham, MA, USA) using magnetic beads coated with anti-CD9, -CD63, and -CD81 antibodies, three tetraspanin proteins highly conserved in the membrane of exosomes. For this, a mix of the three capturing beads was diluted in 500 μL 1X PBS with 2.5% BSA: 10 μL anti-CD9 magnetic beads (10614D; Thermo Fisher Scientific, Waltham, MA, USA), 20 μL anti-CD63 magnetic beads (10606D; Thermo Fisher Scientific, Waltham, MA, USA), and 10 μL anti-CD81 magnetic beads (10616D; Thermo Fisher Scientific, Waltham, MA, USA). Magnetic beads were pre-washed with 500 µL of 1X PBS 2.5% BSA for 5 min. The mix was then washed with 1X PBS 1%-Tween-20 for 5 min. To reduce electrostatic interactions, it was finally rewashed with 1X PBS for 10 min to remove detergent residues; 500 µL of diluted EVs were added on top of the resulting mix of capturing beads and further incubated for 16 h at 8 °C with continuous flicking. After incubation, captured EVs were washed three times with 500 μL 1X PBS 0.001%-Tween-20 and three times with 1X PBS by discarding the supernatant. For the release of captured EVs, the mix of EVs-magnetics beads was incubated with 50 µL of 0.22 µm-filtered glycine 50 mM pH 2.8 for 5 min with continuous mixing at 8 °C, and the solution was neutralized by adding 5 µL of 0.22 µm-filtered Tris-HCl 1M pH 8. Purified EVs were diluted in 450 µL of 0.22 µm-filtered PBS 1X for NTA characterization. To obtain a higher EV concentration, a post-enrichment step was performed using Amicon Ultra-0.5 mL 100 KDa centrifugal filters (Merck KGaA, Darmstadt, Germany): 10 µL were stored at −80 °C for TEM analysis and the remaining volume was stored at 4 °C for WB analysis.

To determine the non-specific background released from magnetic beads by acidic pH, a mix of pre-washed magnetic beads (10 µL anti-CD9 magnetic beads, 20 µL anti-CD63 magnetic beads, and 10 µL anti-CD81 magnetic beads) was incubated overnight with 500 µL of 1X PBS with continuous flicking at 8 °C. The isolated samples were characterized through NTA to determine the non-specific background signal in three replicates, and the average was subtracted from the EV concentration obtained in each bin size from each replicate (N = 6) performed with each EV isolation technique in younger and older adults.

To assess the human specificity of the CD9, CD63, and CD81 antibodies used with magnetic beads, these magnetic beads were incubated with EV samples isolated from cell culture-conditioned media obtained from the SH-SY5Y human neuroblastoma cell line and the RAW 264.7 murine macrophage cell line. EV samples were obtained from 12 mL of cell culture-conditioned media in triplicate using two EV isolation methods: Norgen’s Cell Culture Media Exosome Purification Kit (NORGEN Biotek Corp., Thorold, ON, Canada) and differential UC. Once EVs were isolated, they were incubated for 16 h with a mix of pre-washed magnetic beads with continuous flicking at 8 °C. The isolated samples were characterized through NTA to determine the EV concentration after magnetic bead immunocapture in the human and murine cell lines, separately.

### 2.6. Characterization of Salivary EVs

#### 2.6.1. Nanoparticle Tracking Analysis

The EV concentration (number of particles/mL) and size mode (nm) obtained with each technique, before and after magnetic bead immunocapture, was determined by dynamic light scattering using a NanoSight NS300 system (Malvern Panalytical, Worcestershire, UK).

To achieve a range of 10–100 particles per frame, 100 µL of each salivary EV isolate was diluted with freshly 0.22 µm-filtered 1X PBS to a final volume of 500 µL and loaded into the detection chamber with the NanoSight syringe pump accessory. NTA acquisition settings were maintained identical to characterize EVs obtained before and after magnetic bead immunocapture (blur size: 5 × 5, max jump distance: 13 pixels, minimum track length: 5 consecutive frames, threshold: 2). For each EV sample, five 90-s videos with a minimum of 200 valid tracks/video (minimum of 1000 valid tracks/sample) were recorded at room temperature. The camera level was manually adjusted to achieve optimal visualization of particles, with values ranging from 10 to 12 for each EV sample. Data were processed with the NTA software (version 3.4; Malvern Panalytical, Worcestershire, UK). The concentration of salivary EV isolates was adjusted for the dilution factor applied (1:5).

#### 2.6.2. Western Blot Analysis

For each EV isolation technique, WB analyses were performed using protein extracts from salivary EV samples obtained from 2 mL of cell-free saliva before and after immunocapture with magnetic beads in younger and older adults, respectively. Protein extracts from salivary EVs were obtained using RIPA buffer (Thermo Fisher Scientific, Waltham, MA, USA) with 1X protease inhibitors for 30 min with continuous flicking at 4 °C. The same treatment was applied to the SH-SY5Y human neuroblastoma cell line that was used as a control. Next, 25 µL of each sample was mixed with a non-reducing loading buffer 2X, and boiled at 37 °C for 5 min, 65 °C for 10 min, and 95 °C for 15 min. The same volume was mixed with Laemmli Sample Buffer 2X (Merck KGaA, Darmstadt, Germany) under reducing conditions and boiled at 95 °C for 5 min for denaturation of protein extracts. Samples were separated at either 9% or 13% polyacrylamide gel electrophoresis (SDS-PAGE) and transferred into 0.2 µm nitrocellulose membranes. Membranes were further stained with Ponceau S Staining Solution (Thermo Fisher Scientific, Waltham, MA, USA) to visually control the amount of protein loaded in each lane. To avoid unspecific binding, membranes were blocked for 1 h at room temperature using 5% (*w*/*v*) bovine serum albumin (BSA) in 0.1% 1X Tris-buffered saline-Tween-20 (TBS-T). Primary antibodies were incubated overnight with rotation at 4 °C. The following primary antibodies were used: CD63 (1:250; BD Biosciences, Franklin Lakes, NJ, USA), CD81 (1:250; BD Biosciences, Franklin Lakes, NJ, USA), and flotillin-1 (1:100; BD Biosciences, Franklin Lakes, NJ, USA) as positive EV markers; and GRP78 (1:500; BD Biosciences, Franklin Lakes, NJ, USA) and calnexin (1:1000; Merck KGaA, Darmstadt, Germany) as negative EV markers. The expression of lipoprotein and albumin contamination was further assessed across different EV techniques by using APOB (1:200; Novus Biologicals, Littleton, CO, USA) and albumin (1:1000; Thermo Fisher Scientific, Waltham, MA, USA) antibodies, respectively. The expression of CD63 and CD81 was assessed by SDS-PAGE under non-reducing conditions, whereas the expression of the remaining proteins was assessed under reducing conditions.

Probed membranes were washed three times with TBS-T and incubated with HRP-conjugated secondary antibodies (anti-mouse 1:8000; Thermo Fisher Scientific, Waltham, MA, USA, or anti-rabbit 1:5000; Merck KGaA, Darmstadt, Germany) for 1 h at room temperature with rotation. Next, three washes were performed with TBS-T, and membranes were incubated for a minimum of 1 min with Supersignal West Femto Maximum Sensibility Substrate (Thermo Fisher Scientific, Waltham, MA, USA) to visualize the peroxidase activity. Finally, images were captured and analyzed with an Invitrogen iBright CL1500 imaging system (Thermo Fisher Scientific, Waltham, MA, USA).

#### 2.6.3. Transmission Electron Microscopy

Salivary EVs were imaged using a Zeiss LIBRA 120 transmission electron microscope (Carl Zeiss Microscopy, Thornwood, NY, USA) operating at 80 kV. Briefly, 10 µL of salivary EV samples were layered onto formvar/carbon-coated 300-mesh electron microscopy (EM) grids (Electron Microscopy Sciences, Hatfield, PA, USA) and allowed to settle for 20 min. Grids were then fixed with 0.22 µm filtered 1% glutaraldehyde (*v*/*v*) for 5 min. Afterward, grids were washed with ultrapure water for 2 min and treated with 0.22 µm filtered 2% uranyl acetate in aqueous suspension (*w*/*v*) for 15 min. After removing any excess liquid from the grids by blotting, they were allowed to air-dry overnight at room temperature. Images were captured on a 2 Mpx-SSCCD camera.

For each replicate (N = 6), EV isolation technique (N = 8), magnetic bead immunocapture (pre and post), and age group (younger and older adults), a set of representative TEM images was acquired in the same session. The first two images were captured at low magnification (5000×) with the purpose of checking that EV samples were appropriately adsorbed and negatively stained into the EM grids. Five images were next acquired at 10,000× magnification to manually measure the diameter of single EVs. To distinguish EV morphological features at the highest contrast, higher-magnification images (80,000×) were also captured. All images were acquired at 16-bit integer grayscale, 2048 × 2048 pixel resolution, with scales ranging from 0.39 to 3.6 nm/pixel.

A total of 30 images (6 replicates × 5 images/EV isolation technique) acquired at 10,000× magnification (1.48 × 1.48 nm/pixel) were analyzed manually using the ImageJ software (version 1.43, U.S. National Institutes of Health, Bethesda, MA, USA). Salivary EVs were distinguished from the background and non-EV particles on the basis of their morphology and contrast properties. In general, electron-dense membranous structures with high-contrast edges, rounded or “cup-shaped”, with diameters ranging from ≈30 to 250 nm were considered EVs. EVs located on the edges of the image were excluded when the diameter of such EVs could not be reliably determined.

### 2.7. Practical Aspects Related to Salivary EV Isolation

We next estimated the simplicity of each technique, the time employed for isolating salivary EVs with each technique, and the cost per isolated sample. The estimated parameters were *simplicity of use* (i.e., very easy, easy, or moderately easy), *turn-around time (in hours)* defined as the time taken to complete the EV isolation process in a saliva sample, *hands-on time (in minutes*) defined as the effective working time required to obtain the salivary EV sample, and *cost (in Euros)* to obtain salivary EVs from 500 µL of saliva (the lowest limit of saliva allowed for all techniques). The cost per sample was obtained by dividing the price of each EV isolation kit by the number of 500 µL reactions allowed.

### 2.8. Statistical Analysis

Statistical analyses were performed with the RStudio statistical software (version 1.2.5019). The *ARTool* package (version 0.11.1) was used to perform aligned ranks transformation (ART) ANOVAs, a non-parametric approach employed to evaluate the main and interaction effects between EV isolation techniques, magnetic bead immunocapture, and age on EV concentration (particles/mL), EV size mode (nm), recovered EVs (%), protein concentration (µg/mL), and EV/protein ratio (particle number/µg protein). Statistical models included different crossed random terms depending on the regressor of interest. For the evaluation of the three-way interaction (i.e., age × magnetic bead immunocapture × EV isolation technique), we included the variance associated with the replicates. However, for the evaluation of the main effects (i.e., age, magnetic bead immunocapture, or EV isolation technique) and the two-way interactions (i.e., age × magnetic bead immunocapture, age × EV isolation technique, or magnetic bead immunocapture × EV isolation technique), the variance associated with age, magnetic bead immunocapture, and EV isolation technique was also included in statistical models. Bonferroni-adjusted *p*-values from post hoc comparisons were obtained with the *emmeans* package (version 1.8.4).

The relative frequency distribution of salivary EV size mode obtained from TEM images (30 images/technique = 6 replicates × 5 images) was analyzed for each technique after magnetic bead immunocapture.

The same statistical approach was employed for assessing age-related effects on salivary EVs at the group level in experiment 2, using the EV isolation technique that proved to be the most effective in experiment 1. The only difference with experiment 1 was that the source of variation was the individuals instead of the replicates.

## 3. Results

### 3.1. Assessment of Data Reliability

NTA calibration measurements showed a high agreement between theoretical and measured concentrations using five different dilutions (r = 0.99; *p* < 0.0004), supporting the reliability of particle concentrations obtained in the present study (Supplementary Figure S1A). NTA calibration measurements revealed minor deviations from the 100 nm carboxylated polystyrene particles (CPPs) (mean *±* SD diameter = 101.5 ± 1.3 nm; range: 99.5–103.5 nm) (Supplementary Figure S1B), suggesting that our NTA system accurately estimated the size of monodisperse particles.

The non-specific background associated with elution after magnetic immunocapture was calculated (Supplementary Figure S2) and subsequently subtracted from post-immunocapture signals resulting from each EV isolation technique in younger and older adults, separately. The human specificity of CD9, CD63, and CD81 antibodies used with magnetic beads was also confirmed (Supplementary Figure S3).

All EV isolation techniques employed in the present study achieved salivary EVs from 500 µL of cell-free saliva. Table 1 and Table 2 summarize the characteristics of salivary EVs in younger and older adults, respectively. Table 3 contains practical details regarding each EV isolation technique employed in this study. The NTA characterization of salivary EVs obtained with each technique before and after immunocapture in younger and older adults are shown in Supplementary Figures S4 and S5, respectively.

### 3.2. Salivary EV Concentration

We showed that concentration of salivary EVs was affected by age (F_1,177_ = 12.0, *p* = 0.0007), being higher in older (2.8 × 10^9^ ± 2.3 × 10^9^ particles/mL) than in younger adults (2.0 × 10^9^ ± 1.7 × 10^9^ particles/mL); by magnetic bead immunocapture (F_1,177_ = 172.7, *p* < 10^−15^), with lower values after (1.1 × 10^9^ ± 8.1 × 10^8^ particles/mL) than before magnetic bead recovery (3.7 × 10^9^ ± 2.2 × 10^9^ particles/mL); and by the EV isolation technique (F_7,177_ = 7.6, *p* < 10^−7^), with Invitrogen Total Exosome Isolation Solution and ExoQuick-TC ULTRA techniques showing the highest (3.6 × 10^9^ ± 2.8 × 10^9^ particles/mL) and lowest (1.2 × 10^9^ ± 7.4 × 10^8^ particles/mL) EV yield, respectively.

**Figure 2:**
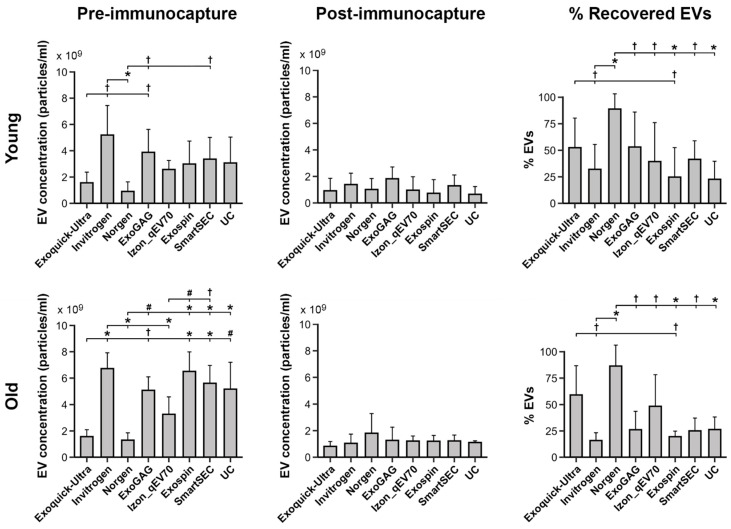
Salivary EV yield obtained with each technique (N = 6 replicates) before and after magnetic bead immunocapture with anti-CD9, -CD63, and -CD81 antibodies (left and middle columns, respectively) in younger and older adults (top and bottom rows, respectively). The right column shows the percentage of recovered salivary EVs obtained with each technique after magnetic bead immunocapture. Due to the lack of significant age × technique interaction on the percentage of recovered salivary EVs, the results of the post hoc analysis for the EV isolation techniques were identical in the two age groups. Statistical differences in EV yield between techniques resulted from post hoc comparisons as long as a significant main or interaction effect was previously obtained with aligned ranks transformation ANOVAs. The *p*-values were adjusted by a Bonferroni correction for multiple comparisons. Statistical significance is expressed as ^†^ (*p* ≤ 0.05); # (*p* ≤ 0.001); * (*p* ≤ 0.0001).

Age further modulated the relationship between magnetic bead immunocapture and EV isolation techniques (F_7,155_ = 3.0, *p* = 0.005). Post hoc analyses derived from the interaction age × magnetic bead immunocapture × EV isolation technique are displayed in Figure 2 (left and middle columns). Importantly, age differences associated with EV isolation techniques were only observed before magnetic bead immunocapture. In this condition, Norgen and ExoQuick-TC ULTRA techniques showed the lowest EV yield in the two age groups. Additionally, age-affected salivary EV concentration obtained with Exo-spin (*p* = 0.003) and SmartSEC (*p* = 0.02), being higher in older (Exo-spin: 6.5 × 10^9^ ± 1.5 × 10^9^ particles/mL; SmartSEC: 5.6 × 10^9^ ± 1.3 × 10^9^ particles/mL) than in younger adults (Exo-spin: 3.0 × 10^9^ ± 1.7 × 10^9^ particles/mL; SmartSEC: 3.3 × 10^9^ ± 1.6 × 10^9^ particles/mL). While the salivary EV yield obtained by UC was statistically unaffected by age, this isolation technique outperformed that obtained by ExoQuick-TC ULTRA (*p* = 0.0009) and Norgen (*p* = 0.0001) in older participants.

Further analysis showed a significant main effect of EV isolation techniques in the percentage of recovered EVs (F_7,83_ = 8.4, *p* < 10^−6^). Overall, Norgen and Exo-spin showed the highest (86.4 ± 18.2) and lowest (21.5 ± 19.4) percentages of recovered EVs, respectively. Neither age nor age × EV isolation technique significantly affected the percentage of recovered EVs. Figure 2 (right column) displays the percentage of recovered EVs for the different EV isolation techniques in younger and older adults, respectively.

### 3.3. Salivary EV Size Mode

**Figure 3:**
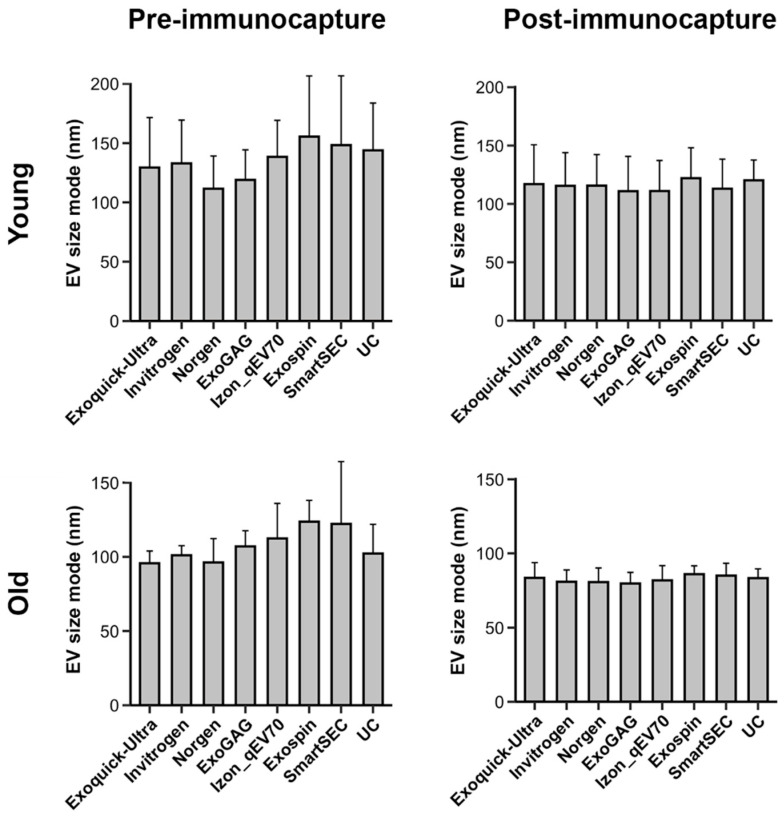
Mean salivary EV size mode obtained with each technique (N = 6 replicates), before and after magnetic bead immunocapture with anti-CD9, -CD63, and -CD81 antibodies (left and right columns, respectively) in younger and older adults (top and bottom rows, respectively). Note that the EV size mode significantly varied with age and magnetic bead immunocapture, but not with EV isolation techniques. nm: nanometers.

Analyses revealed that EV size mode varied with age (F_1,177_ = 103.3, *p* < 10^−15^), being higher in younger (125.3 *±* 34.6 nm) than in older adults (95.2 *±* 20.5 nm), and with magnetic bead immunocapture (F_1,177_ = 61.3, *p* < 10^−12^), showing smaller EV size mode after (99.2 ± 24.9 nm) than before immunocapture (121.3 ± 34.7 nm). However, the EV size mode was statistically unaffected by EV isolation techniques. Figure 3 displays the EV size mode for each age group, the magnetic bead immunocapture condition, and the EV isolation technique. Neither the three-way nor the two-way interactions were statistically significant.

### 3.4. Protein Concentration and Purity of Salivary EVs

**Figure 4:**
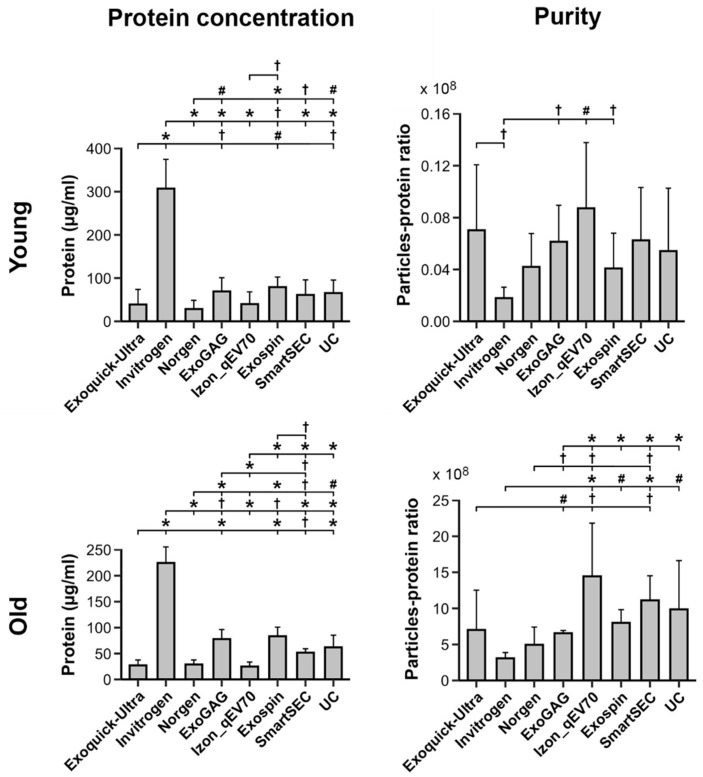
Protein concentration and purity of salivary EVs (left and right columns, respectively) obtained with each technique (N = 6 replicates) before magnetic bead immunocapture with anti-CD9, -CD63, and -CD81 antibodies in younger and older adults (top and bottom rows, respectively). Statistical differences in protein concentration or purity between techniques resulted from post hoc comparisons as long as a significant main or interaction effect was previously obtained with aligned ranks transformation ANOVAs. The *p*-values were adjusted by a Bonferroni correction for multiple comparisons. Statistical significance is expressed as ^†^ (*p* ≤ 0.05); # (*p* ≤ 0.001); * (*p* ≤ 0.0001).

Figure 4 displays the protein concentration and purity of salivary EVs obtained with each technique before magnetic bead immunocapture in younger and older adults, respectively. Analyses showed that protein concentration varied across EV isolation techniques as a function of age (F_7,75_ = 4.1, *p* = 0.0007). The Invitrogen Total Exosome Isolation technique showed the highest level of protein concentration in both younger (306.9 *±* 67.8 µg/mL) and older adults (224.4 ± 31.0 µg/mL). The remaining techniques revealed comparable levels of protein concentration in the two age groups, although the protein concentration exhibited by ExoGAG and Exo-spin was somewhat higher than other techniques in older adults. In contrast, Norgen showed a lower protein concentration than the remaining techniques in the two age groups, with the exception of the ExoQuick-TC ULTRA and Izon qEVOriginal-70nm techniques.

Purity, as revealed by the particle-protein ratio, also varied across EV isolation techniques as a function of age (F_7,75_ = 16.2, *p* < 10^−12^). All techniques showed higher purity in older than in younger adults. Izon qEVOriginal-70nm and Invitrogen Total Exosome Isolation techniques showed the highest (younger: 8.7 × 10^7^ ± 5.1 × 10^7^; older: 1.4 × 10^10^ ± 7.4 × 10^9^) and lowest (younger: 1.8 × 10^7^ ± 8.4 × 10^6^; older: 3.1 × 10^9^ ± 8.1 × 10^8^) mean particle-protein ratios in both age groups, respectively. As illustrated in Figure 4, the purity of Izon qEVOriginal-70nm columns was significantly higher than ExoQuick-TC ULTRA, Invitrogen, Norgen, and ExoGAG, though it was statistically comparable to Exo-spin, SmartSEC, and UC in older participants. Conversely, the Invitrogen Total Exosome Isolation technique showed significantly lower particle-protein ratios than Izon qEVOriginal-70nm, Exo-spin, SmartSEC, and UC in older participants, and lower than ExoQuick-TC ULTRA, ExoGAG, Izon qEVOriginal-70nm and Exo-spin in the younger group.

### 3.5. Protein Characterization of Salivary EVs

**Figure 5:**
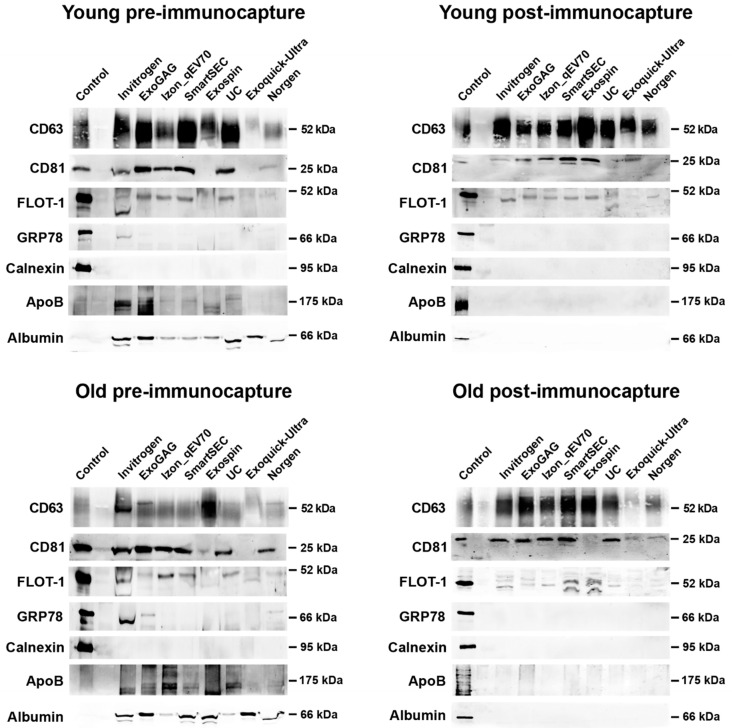
Western blot analyses of salivary EVs obtained with the eight techniques in younger and older adults (top and bottom row, respectively), before and after magnetic bead immunocapture with anti-CD9, -CD63, and -CD81 antibodies (left and right columns, respectively). Antibodies against CD63, CD81, and flotillin-1 (FLOT-1) were used as positive markers of exosomes. As negative markers of exosomes, we used antibodies against Glucose-regulated protein 78 (GRP78) and Calnexin, two endoplasmic reticulum proteins. Levels of lipoprotein and albumin contamination were further assessed by using antibodies against APOB and albumin, respectively. The SH-SY5Y human neuroblastoma cell line was used as a control.

Overall, saliva-derived EVs showed abundant anti-CD63 immunoreactivity, regardless of age, EV isolation technique, and magnetic bead immunocapture (Figure 5). Salivary EVs also exhibited a moderate amount of CD81 and flotillin-1 in the two age groups, although the expression of these two proteins was lower in those techniques that revealed poorer EV yield (i.e., ExoQuick-TC ULTRA and Norgen). The presence of APOB and Albumin observed in younger and older adults became negligible after magnetic bead immunocapture.

### 3.6. TEM Analyses of Salivary EVs

**Figure 6:**
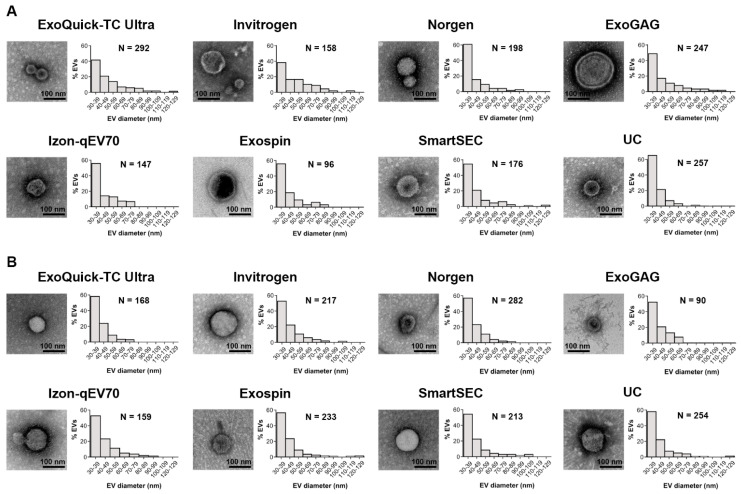
TEM imaging analysis of salivary EVs obtained with each technique after magnetic bead immunocapture with anti-CD9, -CD63, and -CD81 antibodies in younger (**A**) and older adults (**B**). For each technique, we showed a representative negative-stained TEM image at 80,000× magnification together with a histogram displaying the relative frequency distribution of salivary EV size obtained from 30 TEM images (6 replicates × 5 images/technique) acquired at 10,000× magnification. The number of salivary EVs detected with each technique was also included. nm: nanometers.

Figure 6 shows representative TEM images of salivary EVs captured at 80,000× magnification obtained for each technique after magnetic bead immunocapture, together with the relative frequency distribution of measured EV sizes in the two age groups. We analyzed 1571 and 1616 salivary EVs in younger and older adults, respectively, identified in a set of 30 images per technique acquired at 10,000× magnification. Exo-spin and Exoquick-TC Ultra showed the minimum and maximum number of salivary EVs in younger adults (96 and 292, respectively), similar to ExoGAG and Norgen in older adults (90 and 282, respectively). Regardless of the EV isolation technique and age, the mean size of salivary EVs ranged approximately from 30 to 100 nm. About 82% (SD: 5.1, range: 73–89 nm) and 86% (SD: 3.4, range: 81–90 nm) of salivary EVs showed diameters extending from 30 to 60 nm in younger and older adults, respectively. Overall, the highest percentage of salivary EVs emerged in the range of 30–39 nm.

### 3.7. Effects of Age on Salivary EVs: A Group Analysis

**Figure 7:**
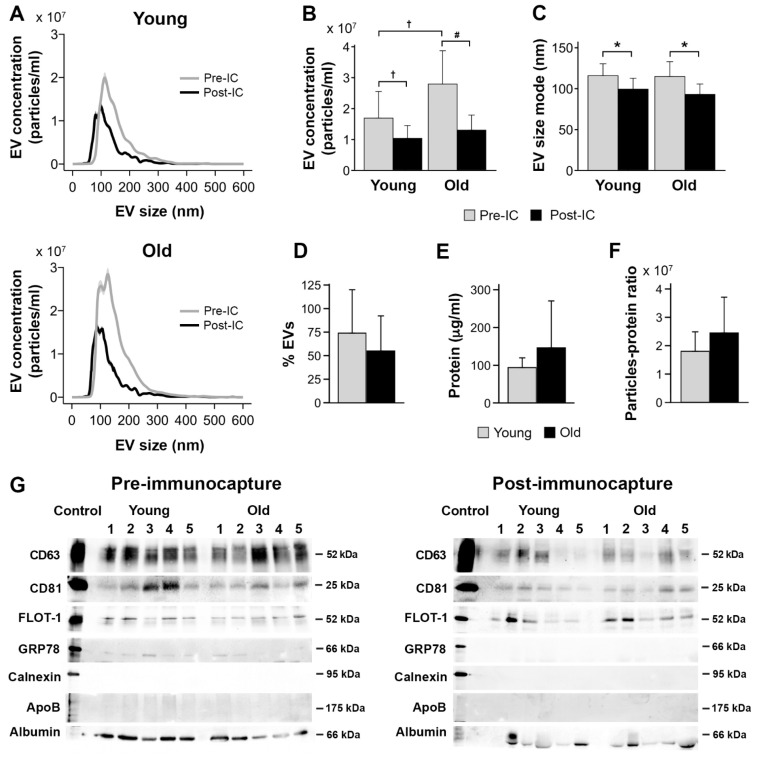
Age effects on salivary EVs obtained with Norgen’s Saliva Exosome Purification Kit comparing 10 younger vs. 10 older adults. (**A**) NTA characterization of salivary EVs before and after magnetic bead immunocapture with anti-CD9, CD63, and CD81 antibodies in younger and older adults. Age differences in salivary EV concentration (**B**), size mode (**C**), percentage of recovered EVs after magnetic bead immunocapture (**D**), level of protein concentration estimated with BCA before magnetic bead immunocapture (**E**), salivary EV purity estimated with the particle-to-protein ratio before magnetic bead immunocapture (**F**), and western blot analyses of salivary EVs obtained before (left panel) and after magnetic bead immunocapture (right panel) in 5 younger and 5 older adults (**G**). Statistical significance is expressed as ^†^ (*p* ≤ 0.05); # (*p* ≤ 0.01); * (*p* ≤ 0.001). IC: immunocapture; nm: nanometers.

While Norgen and ExoQuick-TC ULTRA achieved the highest percentage of salivary EV recovered after magnetic bead immunocapture and the lowest protein concentration, Norgen outperformed ExoQuick-TC ULTRA on turn-around time and cost per sample (Table 3). Based on these findings, we assessed the reproducibility of results obtained with Norgen’s Saliva Exosome Purification Kit in a sample of 10 younger and 10 older participants. Figure 7 displays the results of this experiment. Analyses corroborated that salivary EV yield obtained with Norgen was affected by age (F_1,18_ = 6.0, *p* = 0.02) and magnetic bead immunocapture (F_1,19_ = 22.7, *p* = 0.0001) (Figure 7A). More specifically, the concentration of salivary EVs was higher in older than in younger adults (older: 2.0 × 10^9^ ± 1.1 × 10^9^ particles/mL, younger: 1.4 × 10^9^ ± 7.4 × 10^8^ particles/mL), and higher before than after immunocapture (before: 2.2 × 10^9^ ± 1.1 × 10^9^ particles/mL, after: 1.2 × 10^9^ ± 4.6 × 10^8^ particles/mL). The interaction age × magnetic bead immunocapture was also significant (F_1,18_ = 4.8, *p* = 0.04), revealing that older adults showed higher EV yield than younger participants before magnetic bead immunocapture (older: 2.8 × 10^9^ ± 1.1 × 10^9^ particles/mL, younger: 1.7 × 10^9^ ± 8.6 × 10^8^ particles/mL) (Figure 7B). The magnetic bead immunocapture varied the size mode of salivary EVs (F_1,19_ = 22.4, *p* = 0.0001), being smaller after than before immunocapture (before: 115.4 *±* 16 nm, after: 96.3 *±* 13 nm) (Figure 7A,C). Neither age nor the interaction age × magnetic bead immunocapture affected the size mode of salivary EVs. The percentage of recovered EVs was unaffected by age (Figure 7D). Both protein concentration and purity of salivary EVs obtained with Norgen were statistically comparable in the two age groups (Figure 7E,F).

Figure 7G shows the protein characterization of salivary EVs isolated by Norgen. Salivary EVs showed immunoreactivity to anti-CD63, CD81, and flotillin-1 antibodies in younger and older adults, which was markedly attenuated after magnetic bead immunocapture. The lack of signal from GRP78 and calnexin suggests that salivary EVs obtained with Norgen are likely free of cellular contaminants, although a weak detection of GRP78 was still observed before immunocapture in some cases. Lipoprotein expression, as revealed by APOB detection, were negligible in the two age groups. Albumin contamination was evident in younger and older adults, suggesting that salivary EVs co-elute with albumin even after immunocapture, that albumin is part of the cargo of salivary exosomes, and/or that the albumin signal is intrinsic to the immunocapture processing.

## 4. Discussion

Salivary EVs have immense potential as biomarkers of non-communicable chronic diseases [26,27,28], due to their non-invasive and relatively simple collection methods and to the ability of EVs to cross epithelial barriers [29,30,31] and reach the salivary glands via peripheral blood [32]. However, the lack of studies assessing the efficiency and purity of different EV isolation techniques hinders the use of salivary EVs in clinical settings. Here, we provide the first comparative study of different isolation and purification techniques for human salivary EVs in younger and older adults. The results of this study represent a step forward for including salivary EVs in standardized protocols aimed at establishing the value of salivary EVs in the prognosis, diagnosis, and therapeutic intervention of tissue-specific diseases.

The results revealed a higher concentration of salivary EVs in older adults compared to the younger group, although this effect vanished after the immunocapture with three tetraspanin EV markers. One possible interpretation for this finding is that, in older adults, a significant subset of salivary EVs has a low affinity for the tetraspanin EV markers employed in the present study. Alternatively, further non-exosome particles may coexist with salivary EVs in the elderly. In this regard, circulating bacterial EVs have been shown to increase with age in humans and mice due to an age-related increase in intestinal permeability [33], which is in turn shaped by lifestyle factors such as dietary habits, smoking, level of physical exercise, antibiotic use, sleep quality, and depression [34]. Both interpretations should be specifically evaluated in further experiments.

The integrity of the immune system tends to deteriorate with age, leading to chronic, low-grade inflammation that ultimately increases susceptibility to infections and tissue degeneration [35,36]. Chronic systemic inflammation/infection in the oral cavity is indeed considered an independent risk factor for accelerated aging, aging-related diseases, and mortality [37]. Previous studies have shown that human salivary exosomes contain immunoglobulin A (IgA) and dipeptidyl peptidase IV [12,38], two major elements playing a regulatory role in local immune defense in the oral cavity. Aging-related changes in salivary EVs may also result from a compensatory response of the adaptive immune system to defend against the chronic persistence of pathogens due to aging-related conditions affecting salivary glands such as xerostomia (i.e., subjective sensation of oral dryness) and/or periodontal disease, a chronic inflammatory condition that increases its prevalence with age. However, our results contrast with those obtained from plasma-derived exosomes. Thus, in a cross-sectional study performed on community-dwelling individuals (ages ranging from 30 to 64 years), the plasmatic EV concentration, but not the EV size, declined with age [39]. In line with this, plasmatic EV mitochondrial DNA levels have also been shown to be reduced in an age-dependent manner [40]. Age-related decreased concentrations of plasma EVs may result from impaired clearance mechanisms, dysfunction of proteostasis, cellular senescence, and/or altered intercellular signaling; all these processes are disturbed with aging [41], and they could alter the release of plasmatic EVs to peripheral blood.

Norgen and ExoQuick-TC ULTRA outperformed the remaining techniques in the percentage of salivary EVs recovered after magnetic bead immunocapture. Conversely, these two techniques also exhibited the lowest salivary EV yield, which may be a limitation depending on the final application of the study. Although both techniques use a polymer-based reagent to precipitate and further purify EVs, their differences in the isolation of salivary EVs should be recognized. While both techniques were statistically comparable in terms of protein concentration and purity, Norgen showed somewhat higher levels in CD63 and CD81 proteins compared to ExoQuick-TC ULTRA, suggesting that Norgen presents more affinity for subpopulations of EVs carrying these tetraspanin EV markers. These differences were accompanied by a dissimilar size distribution of single salivary EVs, as revealed by TEM analyses. Remarkably, Norgen showed a higher percentage of smaller EVs (i.e., 30–50 nm) compared to ExoQuick-TC ULTRA, likely revealing that Norgen was better at capturing salivary exosome II than ExoQuick-TC ULTRA. Salivary exosome II is smaller and contains higher levels of dipeptidyl peptidase IV than salivary exosome I [12,38]. Last but not least, ExoQuick-TC ULTRA took a higher turnaround time, due to the overnight incubation step, and the cost of reagents per sample was higher compared with Norgen (Table 3).

We found that Izon qEVOriginal-70nm columns showed the lowest protein concentration and the highest purity, confirming previous evidence that SEC-based methods outperform other EV isolation techniques in terms of purity of the isolated exosomes [42]. The lower protein concentration of Izon qEVOriginal-70nm columns came at the expense of overall EV yield and cost per sample, despite these columns can be washed and reused several times. In contrast, the Invitrogen Total Exosome Isolation method showed the highest level of protein concentration and the lowest purity, likely hindering further analysis of salivary EVs for downstream applications. Interestingly, Norgen and ExoQuick-TC ULTRA showed comparable levels of protein concentration to Izon qEVOriginal-70nm columns in the two age groups, which may account for the composition of reagents. Norgen uses silicon carbide (SiC) to isolate EVs from biofluids [43], leading to lower levels of protein concentration [44], a higher yield of exosomal marker proteins and RNA, and an increased reproducibility of results [45]. Alternatively, ExoQuick-TC ULTRA is a polymer-based method containing polyethylene glycol (PEG) species with a molecular weight of 8000 Daltons [46]. PEG is a hydrophilic volume-excluding polymer that wraps around water molecules and forces less soluble particles to precipitate [47], allowing large amounts of EVs without significant sacrifice in purity [48]. Norgen and ExoQuick-TC ULTRA are reasonable alternatives in terms of purity of salivary EV samples, although turnaround time and reagent cost per sample are notably higher in ExoQuick-TC ULTRA compared to Norgen.

Previous studies have shown that salivary EVs contain a variety of exosome markers [13,16,18,38], particularly CD63, CD81, CD9, Alix/AIP1, and TSG101. Here, we corroborated that salivary EVs showed high expression of CD63 and moderate immunoreactivity to anti-CD81 and anti-flotillin-1 antibodies, suggesting that these particles can be considered exosomes. The CD63 enrichment was maintained across all EV isolation techniques and age groups, whereas CD81 and flotillin-1 were more inconsistently expressed, probably due to the lower salivary EV yield obtained with some techniques (e.g., ExoQuick-TC ULTRA and Norgen). Tetraspanins, such as CD63 and CD81, are highly enriched on exosomes [49], play a pivotal role in stabilizing the exosome structure [50], and seem to be required to dock exosomes to the surface of target cells [51]. However, tetraspanins are heterogeneously detected in exosomes from different cell lines [52] and biofluids [53], likely reflecting dissimilar expression levels of their secretory cells. We speculate that the abundant CD63 and CD81 expression detected in salivary exosomes may indicate their participation in the immune response in the oral cavity [54,55]. Salivary exosomes were also verified with the presence of flotillin-1. Flotillins are ubiquitous and highly conserved proteins that directly regulate the formation of Cadherin complexes at plasma membrane microdomains, thereby allowing the formation of cell-cell contact sites [56,57]. Flotillins also contribute to the targeted delivery of membrane proteins from the intracellular compartments to very specific sites in a cell type-specific manner [58]. In the salivary glands, flotillins make use of Clathrin-independent endocytosis for the internalization of the Muscarinic type 3 receptor [59], a G-protein-coupled receptor located in the plasma membrane involved in saliva secretion [60]. Accordingly, the expression of flotillin-1 in salivary exosomes may reflect the integrity of salivary glands, which is indirectly supported by the lower immunoreactivity of flotillin-1 in older compared to younger adults.

Preliminary work revealed the presence of two types of exosomes in human saliva (exosomes I and II) differing in their size and protein composition [38]. Exosomes II are smaller (mean diameter: 41 nm, range: 20 to 80 nm) and more abundant than exosomes I (mean diameter: 84 nm, range: 30 to 250 nm). Exosomes I and II contain high levels of immunoglobulin A (IgA), suggesting that both types of salivary exosomes play a major role in local immune defense in the oral cavity. Although the present study cannot discriminate between both types of salivary exosomes, we showed that EV isolation techniques are mostly able to detect salivary EVs of diameters ranging from 30 to 60 nm, as revealed by morphometric analysis of TEM images. Remarkably, NTA characterization performed after magnetic bead immunocapture showed that salivary EV size mode from older adults was, on average, about 32 nm lower (mean: 83 nm, range: 80 to 86 nm) than those obtained from younger participants (mean: 115 nm, range: 102 to 122 nm). As the salivary EV yield obtained after magnetic bead immunocapture was unaffected by age, size differences in salivary EVs with age may reflect an adaptive response to aging-related increased susceptibility to infections in the oral cavity. Further research is required to elucidate whether aging affects the distribution and cargo of the two salivary exosome types and ascertain the biological meaning of these changes.

This study has some limitations that should be acknowledged. While the spitting technique has obvious advantages for saliva collection, saliva specimens are not strictly unstimulated since this technique may have some stimulatory effects [24]. Analyses aimed at comparing different EV isolation techniques were performed with saliva obtained from a small sample (3 younger and 3 older adults). Therefore, results should be interpreted carefully and replicated with larger cohorts. Given the large volumes of saliva required for this study, saliva samples were pooled for each age group. This approach allowed us to include a number of replicates to use non-parametric statistical analysis methods at the cost of impeding the estimation of the inter-individual variability in each technique. Finally, immunocapture of salivary EVs with anti-CD9, -CD63, and -CD81 antibodies may exclude other subtypes of salivary EVs (e.g., bacterial EVs) useful for determining different aging trajectories or risk levels for aging-related diseases. All these limitations should be addressed in future experiments.

## 5. Conclusions

We have comprehensively compared eight techniques for the isolation of human salivary EVs before and after immunocapture with magnetic beads. The effects of age on the concentration, size, and purity of salivary EV were also assessed. Results may contribute to the standardization of salivary EV protocols and the discovery and validation of potential biomarkers of systemic diseases in salivary EVs across the lifespan.

## Figures and Tables

**Table 1:** Summary of salivary EV measurements in younger adults.

EV Isolation Technique	EV Concentration (Particles/mL)		EV Size Mode (nm)		Recovered EVs (%)	Protein (µg/mL)	Purity
	Pre-MBI	Post-MBI	Pre-MBI	Post-MBI			
ExoQuick-TC Ultra	1.6 × 10^9^ ± 8.1 × 10^8^	8.3 × 10^8^ ± 5.9 × 10^8^	129 ± 42	117 ± 33	57 ± 27	39 ± 35	7.0 × 10^7^ ± 5.0 × 10^7^
Invitrogen	5.2 × 10^9^ ± 2.2 × 10^9^	1.5 × 10^9^ ± 8.8 × 10^8^	133 ± 36	115 ± 28	34 ± 25	307 ± 68	1.8 × 10^7^ ± 8.4 × 10^6^
Norgen	9.0 × 10^8^ ± 7.3 × 10^8^	8.9 × 10^8^ ± 7.4 × 10^8^	111 ± 28	116 ± 27	97 ± 2	28 ± 20	4.2 × 10^7^ ± 2.6 × 10^7^
ExoGAG	3.9 × 10^9^ ± 1.7 × 10^9^	1.7 × 10^9^ ± 6.0 × 10^8^	119 ± 25	111 ± 30	55 ± 33	69 ± 32	6.1 × 10^7^ ± 2.8 × 10^7^
Izon qEV70	2.6 × 10^9^ ± 6.9 × 10^8^	1.0 × 10^9^ ± 9.5 × 10^8^	138 ± 31	102 ± 16	42 ± 36	40 ± 28	8.7 × 10^7^ ± 5.1 × 10^7^
Exo-spin	3.0 × 10^9^ ± 1.7 × 10^9^	8.0 × 10^8^ ± 1.0 × 10^9^	155 ± 50	122 ± 26	28 ± 28	79 ± 23	4.1 × 10^7^ ± 2.7 × 10^7^
SmartSEC	3.4 × 10^9^ ± 1.7 × 10^9^	1.4 × 10^9^ ± 8.5 × 10^8^	148 ± 58	113 ± 25	45 ± 19	61 ± 35	6.2 × 10^7^ ± 4.1 × 10^7^
UC	3.1 × 10^9^ ± 2.0 × 10^9^	7.2 × 10^8^ ± 5.9 × 10^8^	144 ± 40	120 ± 17	25 ± 17	65 ± 30	5.4 × 10^7^ ± 4.8 × 10^7^

EV: Extracellular vesicles; MBI: Magnetic bead immunocapture; UC: differential ultracentrifugation.

**Table 2:** Summary of salivary EV measurements in older adults.

EV Isolation Technique	EV Concentration (Particles/mL)		EV Size Mode (nm)		Recovered EVs (%)	Protein (µg/mL)	Purity
	Pre-MBI	Post-MBI	Pre-MBI	Post-MBI			
ExoQuick-TC Ultra	1.6 × 10^9^ ± 5.3 × 10^8^	8.5 × 10^8^ ± 3.1 × 10^8^	96 ± 8	83 ± 10	59 ± 28	27 ± 10	7.0 × 10^9^ ± 5.5 × 10^9^
Invitrogen	6.7 × 10^9^ ± 1.2 × 10^9^	1.1 × 10^9^ ± 7.0 × 10^8^	101 ± 6	81 ± 8	16 ± 7	224 ± 31	3.1 × 10^9^ ± 8.1 × 10^8^
Norgen	1.3 × 10^9^ ± 5.6 × 10^8^	1.1 × 10^9^ ± 5.1 × 10^8^	96 ± 16	81 ± 9	86 ± 20	29 ± 9	4.9 × 10^9^ ± 2.5 × 10^9^
ExoGAG	5.1 × 10^9^ ± 1.0 × 10^9^	1.3 × 10^9^ ± 1.0 × 10^9^	107 ± 11	80 ± 7	26 ± 17	78 ± 18	6.6 × 10^9^ ± 3.8 × 10^8^
Izon qEV70	3.3 × 10^9^ ± 1.3 × 10^9^	1.3 × 10^9^ ± 4.0 × 10^8^	112 ± 24	82 ± 10	48 ± 30	25 ± 9	1.4 × 10^10^ ± 7.4 × 10^9^
Exo-spin	6.5 × 10^9^ ± 1.5 × 10^9^	1.3 × 10^9^ ± 4.4 × 10^8^	124 ± 14	86 ± 6	19 ± 5	83 ± 18	8.0 × 10^9^ ± 1.8 × 10^9^
SmartSEC	5.6 × 10^9^ ± 1.3 × 10^9^	1.3 × 10^9^ ± 4.5 × 10^8^	122 ± 42	85 ± 8	25 ± 12	51 ± 8	1.1 × 10^10^ ± 3.4 × 10^9^
UC	5.2 × 10^9^ ± 2.0 × 10^9^	1.2 × 10^9^ ± 1.3 × 10^8^	102 ± 20	83 ± 6	26 ± 12	62 ± 24	9.9 × 10^9^ ± 6.8 × 10^9^

EV: Extracellular vesicles; MBI: Magnetic bead immunocapture; UC: differential ultracentrifugation.

**Table 3:** Practical aspects of techniques for the isolation of salivary EVs.

EV Isolation Technique	#Reactions/Volume	Ease-of-Use	Turn-Around Time (h)	Hands-On Time (min)	Cost (€)
ExoQuick-TC Ultra	20/0.5–5 mL	•	12.7	20	35
Invitrogen	24/0.5 mL	•••	2.2	8	18
Norgen	50/0.5–2 mL	••	0.8	25	18
ExoGAG	20/0.5 mL	•••	0.7	8	22
Izon qEV70	^†^ 5/0.5 mL	••	1.1	8	48
Exo-spin	24/0.5–5 mL	•	14	42	20
SmartSEC Single	10/0.5 mL	••	1.2	16	54
UC	N/A	••	3.6	50	5

#Reactions/volume: number of reactions per kit/volume of sample allowed with each reaction. Ease-of-use: simplicity of technology. ••• = very easy; •• = easy; • = moderately easy. Turn-around time: time (in hours) to complete the EV isolation process. Hands-on time: Effective working time (in minutes) to obtain the salivary EV pellet. Cost: Approximate cost to obtain salivary EVs from 500 ul saliva. ^†^ The Izon qEVOriginal-70nm columns can be reused up to 5 times with the same sample. N/A: not applicable. h: hours; min: minutes.

## Data Availability

The datasets employed in the current study are available from the corresponding author upon reasonable request.

## Supplementary Materials

The following supporting information can be downloaded at: https://www.mdpi.com/article/10.3390/cells13010095/s1.

## List of Abbreviations

ANOVA	analysis of variance
BCA	bicinchoninic acid protein assay
CPPs	carboxylated polystyrene particles
EVs	extracellular vesicles
IgA	immunoglobulin A
MiRNAs	microRNAs
NTA	nanoparticle tracking analysis
PEG	polyethylene glycol
SEC	size exclusion chromatography
SiC	silicon carbide
TEM	transmission electron microscopy
UC	ultracentrifugation
WB	western blots
