# Supplementary material for: Characterization of Extracellular Vesicles from Human Saliva: Effects of Age and Isolation Techniques
Source: Cells. 2024 Jan 2;13(1):95. doi: 10.3390/cells13010095 (PMC10778510; doi:10.3390/cells13010095)
Supplement: Supplementary file 1 [file cells-13-00095-s001.zip › cells-2742500-supplementary.pdf]

Characterization of Extracellular Vesicles from Human Saliva: Effects of Age and Isolation Techniques

Lucia Reseco[1,2 †], Angela Molina-Crespo[1,2 †], Mercedes Atienza[1,2], Esperanza Gonzalez[3], Juan Manuel Falcon-Perez[3,4], Jose L. Cantero[1,2*]

[1] Laboratory of Functional Neuroscience, Pablo de Olavide University, 41013 Seville, Spain

[2] CIBERNED, Centro de Investigación en Red de Enfermedades Neurodegenerativas, Instituto de Salud Carlos III, 28029 Madrid, Spain.

[3] Exosomes Laboratory, Center for Cooperative Research in Biosciences (CIC bioGUNE), Basque Research and Technology Alliance (BRTA), 48160 Derio, Spain

[4] CIBEREHD, Centro de Investigación Biomédica en Red de Enfermedades Hepáticas y Digestivas, 28029 Madrid, Spain

* Correspondence: jlcanlor@upo.es

[†]These authors contributed equally to this work.

SUPPLEMENTARY MATERIAL

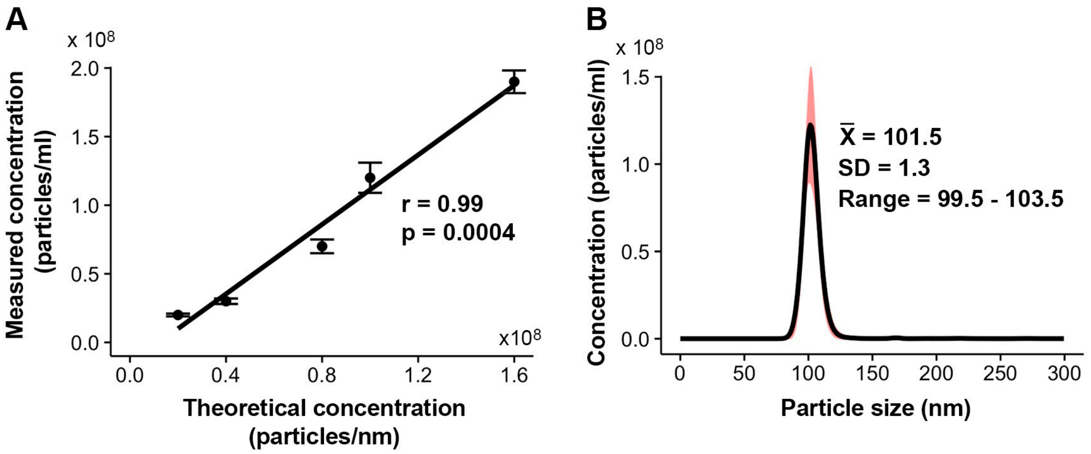

Supplementary Figure S1. NTA calibration of particle concentration and size. **A.** Correlation between theoretical and known particle concentrations obtained at five dilutions (i.e., 1:15,000; 1:22,500; 1:30,000; 1:60,000 and 1:120,000) with 3 replicates per dilution. **B.** Deviations of particle size were negligible when using monodisperse 100 nm carboxylated polystyrene particles (CPPs) at 1:15,000 dilution. Both results support the accuracy of NTA measurements performed in this study.

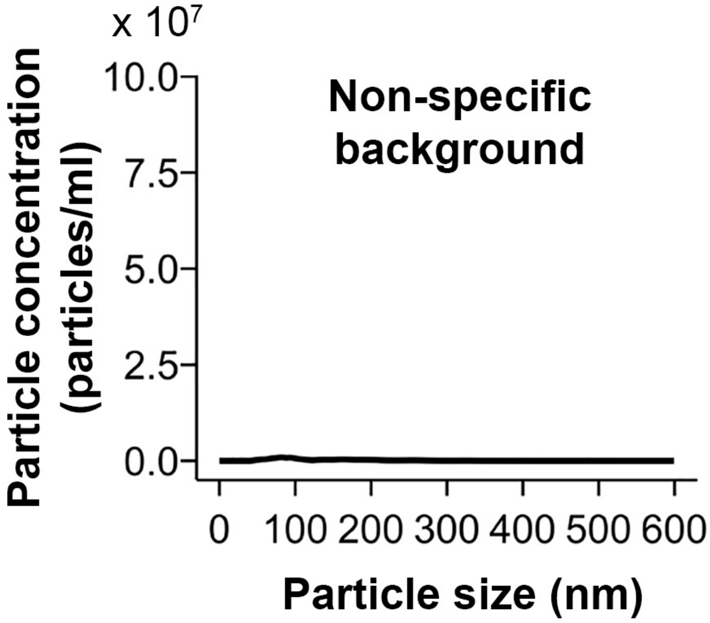

Supplementary Figure S2. Non-specific background signal when particles were eluted from magnetic beads by pH acidification. This non-specific background was subtracted, for each bin size (in nm), from each experiment performed in the present study (N = 3 replicates).

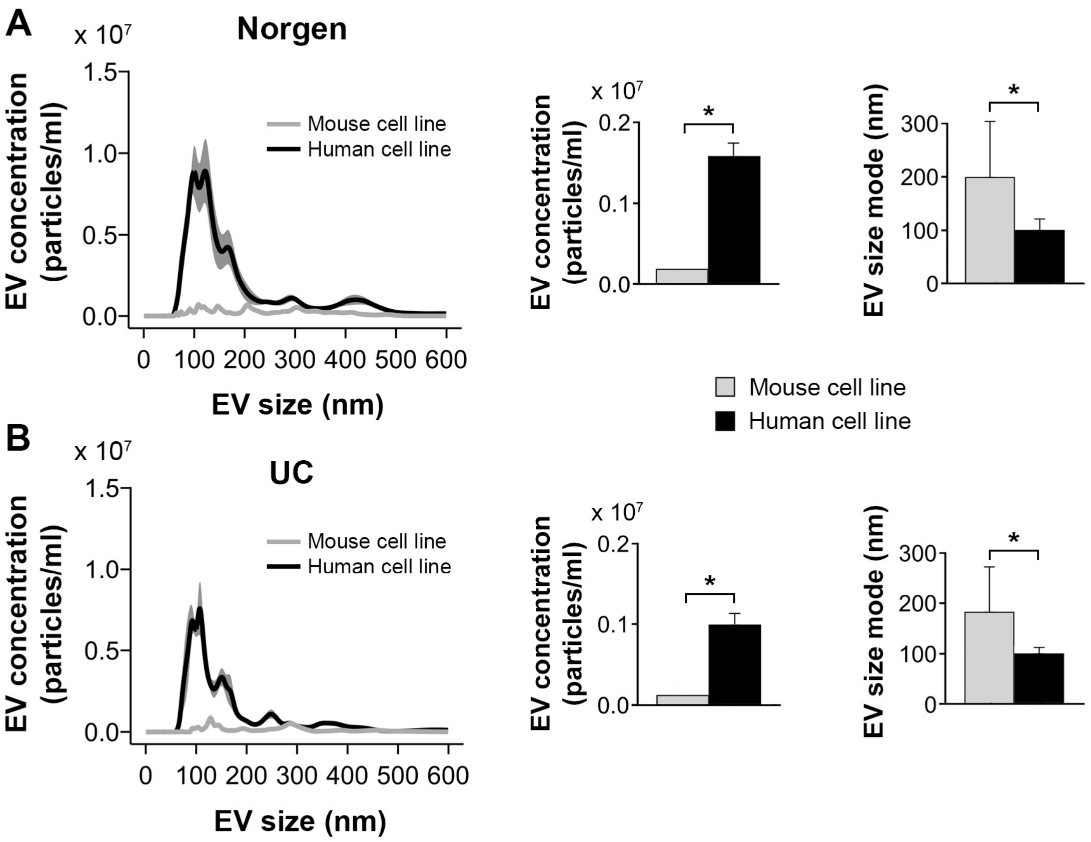

Supplementary Figure S3. NTA characterization of EVs obtained from the SH-SY5Y human neuroblastoma cell line and the RAW 264.7 murine macrophage cell line after magnetic bead immunocapture. Two different EV isolation techniques were employed: Norgen's Cell Culture Media Exosome Purification Kit **(A)** and UC **(B)**, and the EV samples obtained were further immunocaptured using anti-CD9, -CD63, and -CD81 antibodies. The non-specific background was previously subtracted, for each bin width, from each trial (N = 3 replicates) performed with each EV isolation technique. Note that EV concentration resulting from the murine cell line appeared negligible compared to that derived from the human cell line. In contrast to particles obtained from the murine cell line, the mean size mode of particles obtained from the human cell line is in the range of true exosomes. Statistical significance was expressed as * ($p \leq 0.001$).

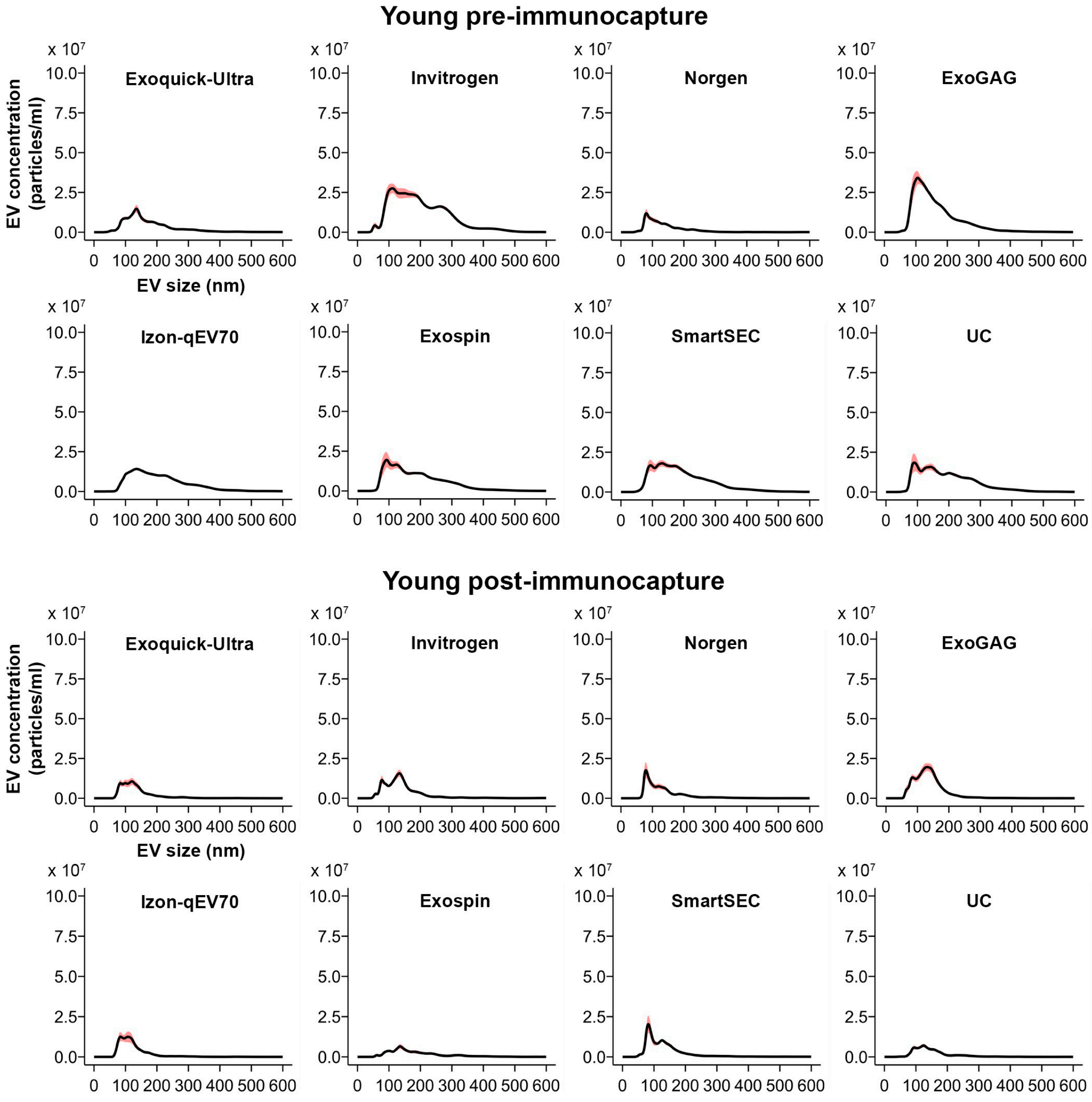

Supplementary Figure S4. NTA characterization of salivary EVs obtained from pooled saliva of three younger adults with each isolation technique before and after immunocapture. Each plot displays the mean (black line) and standard deviation (red line) obtained in each technique (N = 6 replicates). nm: nanometers.

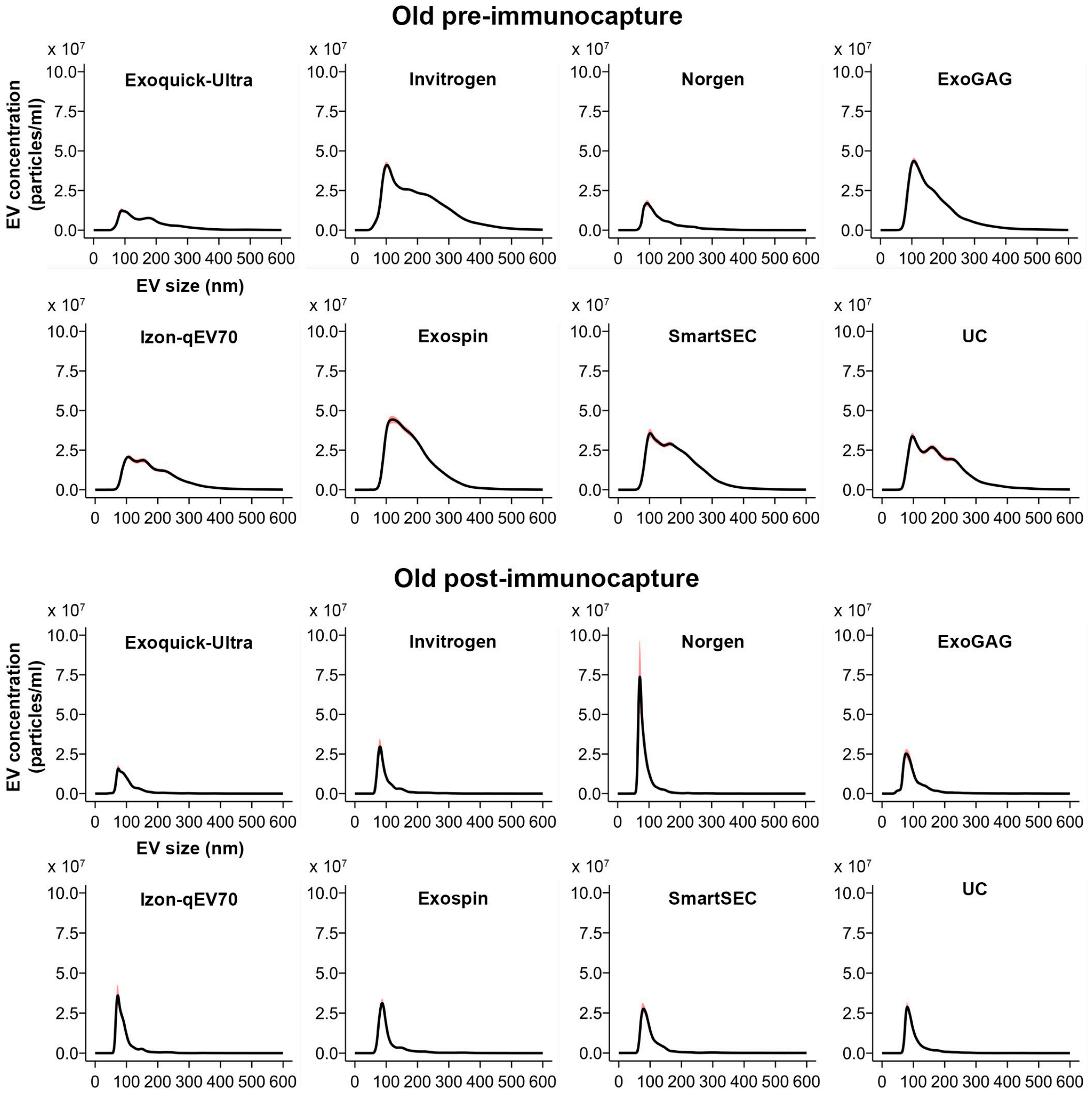

Supplementary Figure S5. NTA characterization of salivary EVs obtained from pooled saliva of three older adults with each isolation technique before and after immunocapture. Each plot displays the mean (black line) and standard deviation (red line) obtained in each technique (N = 6 replicates). nm: nanometers.
